# Interaction of microglia and T cells: a deadly duo behind Tau-mediated neurodegeneration

**DOI:** 10.1038/s41392-023-01563-9

**Published:** 2023-08-21

**Authors:** Bilge Askin, Susanne Wegmann

**Affiliations:** 1 German Center for Neurodegenerative Diseases, Charitéplatz 1, 10117 Berlin, Germany; 2 Einstein Center for Neurosciences Berlin, Charité - Universitätsmedizin Berlin, Berlin, Germany

**Keywords:** Diseases of the nervous system, Immunology

A recent study by Chen et al. published in Nature reported the presence of activated T cells in the brains of transgenic mice with frontotemporal dementia (FTD)-like Tau protein pathology and in postmortem Alzheimer’s disease (AD) brains.^1^ The infiltration of T cells correlated with the degree of Tau pathology and microglia activation, and appeared to promote neurotoxicity.

The progressive aggregation of amyloid-β in extracellular amyloid plaques and of Tau in neurofibrillary tangles are the two main protein pathological hallmarks of AD. They are accompanied by synaptic loss, neuroinflammation, and neuronal death, and compromise the blood-brain barrier, allowing immune cells to infiltrate the brain parenchyma and interact with glial cells and neurons; all of these factors contribute to disease progression.^2^ Activated T cells can be found in postmortem brains of different neurological disorders, in AD, particularly in areas with neuronal loss and Tau pathology, i.e., the hippocampus and limbic structures. This suggests a link between Tau, T cell accumulation, and neurodegeneration.^3^

Chen et al. used single-cell RNA sequencing (scRNA-seq) to systematically investigate the brain immune cell niche in mouse models representing different aspects of AD brain pathology. They studied the immune cell composition in mice that express human FTD-mutant TauP301S and develop Tau tangle pathology, neuroinflammation (microglia and astrocyte activation), and neuronal loss in the hippocampal formation and other parts of the brain (TE4 line, derived from PS19 line), and in mice that develop amyloid plaque pathology (A/PE4 or 5xE4 lines, derived from APP/PS1 and 5xFAD lines) that show less neuronal damage and milder neuroinflammation. Notably, the authors had created these mouse models on human APOE4 and APOE3 knock-in backgrounds to test for the influence of this strong genetic AD risk factor.

In CD45^+^ immune cells isolated from brain parenchyma, or from meninges, 12 types of innate and adaptive immune cells were identified. Interestingly, the composition of meninges immune cells was similar across mouse models, whereas the number of activated T cells (CD4^+^ and CD8^+^) in the parenchyma was substantially higher in mice with Tau pathology compared to mice with A-beta plaques or no pathology (control E4 line); CD8^+^ T cells were especially enriched in Tau mouse brains. This observation was independent of the APOE background (APOE4 (TE4) or APOE3 (TE3)) and correlated with elevated microglia number and neuronal loss. Interestingly, Tau transgenic mice on an APOE knockout background were protected against T cell infiltration, suggesting that the presence but not the isoform of APOE is relevant for this process. In AD brains, T cell infiltration occurred in regions presenting Tau pathology (AT8^+^ neurons) and correlated with Tau pathology progression (Braak staging). These observations suggested that Tau pathology but not A-beta or APOE genotype is responsible for T cell infiltration. APOE may be generally involved in the underlying signaling or glia activation though.

**Fig. 1:**
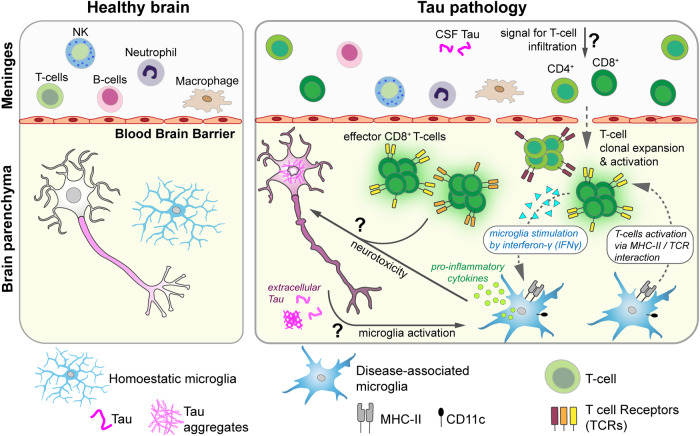
Pathological Tau aggregation triggers the neurotoxic interplay between microglia and infiltrated T cells in the brain. Tau pathology in AD and tauopathies triggers a complex neuroinflammatory response, in which the interaction between activated microglia and effector-type T cells mediates neurotoxicity. In a proposed model, neuronal Tau aggregation triggers microglia activation through an unknown mechanism, resulting in recruitment, clonal expansion, and activation of T cells in the brain parenchyma. Microglial MHC-II and CD11c presentation can activate T cells and be stimulated by IFNγ, for example released by T cells and other immune cells in the brain. Removal of microglia or T cells, or suppressing their activation and IFNγ-based communication, can break this feed-forward cycle of neuroinflammation and reduces neurotoxicity in the context of Tau pathology. Parts of this figure have been created using Biorender

For a closer look at the T cell repertoire in tauopathy mice, the authors performed a detailed analysis of their gene expression signatures. In meninges, naïve CD8^+^ T cells and regulatory T cells were abundant, while effector CD8^+^ T cells were enriched in the parenchyma. The activation of naive T cells upon antigen exposure involves their clonal expansion, resulting in a T cell population with distinct T cell receptors (TCRs) that regulate the adaptive immune response.^4^ Single-cell TCR sequencing (scTCR-seq) revealed that CD4^+^ and CD8^+^ T cells from brains with Tau pathology had clonally expanded and transitioned from an “active” to “exhausted” state with progression of Tau pathology and neuroinflammation. These findings suggest the presence of chronic antigen stimulation in tauopathy mice. Whether extracellular Tau itself can function as an antigen or triggers the presentation of T cell-activating antigens (by neurons or glia cells), remains to be elucidated (Fig. 1).

How are T cells activated in the brains of tauopathy mice?—Chen et al. found a potential explanation for this in the interplay between infiltrated T cells and microglia, the brain-residing immune cells. In microglia from tauopathy mice, genes associated with antigen representation, complement and interferon response, lysosomal pathways, and oxidative stress were upregulated. Furthermore, microglia had an antigen-presenting phenotype^2^ characterized by the expression of major histocompatibility complex, MHC-II, proteins, and CD11c receptor, a disease-associated marker in TREM2^+^ microglia, which can provide a signal for T cell activation. Indeed, primary mouse microglia co-cultured with OT-1T cells (CD8^+^ T cells expressing TCRs that recognize ovalbumin as antigen) were able to stimulate OT-1T proliferation in the presence of their antigen, ovalbumin, which was exacerbated upon stimulation of microglia with the pro-inflammatory cytokine interferon-gamma, IFNγ. Inhibiting IFNγ signaling, or removing microglia by PLX3397 administration reduced p-Tau accumulation and brain atrophy in tauopathy mice. These results show that activated microglia have the capacity to trigger a T cell response that promotes Tau pathology and neurotoxicity. IFNγ secretion by immune cells could feed this interplay and exacerbate neurodegeneration in tauopathies. Importantly, amyloid plaque pathology did not induce this cascade, indicating that human Tau accumulation, phosphorylation, aggregation, or release (all occurring in tauopathy mouse models and AD patients) trigger a unique microglia reaction. How neurons signal such Tau alterations to microglia and induce T cell infiltration and activation remains to be clarified. This may, for example, involve the presentation of neuronal surface cues in response to intracellular Tau aggregation^5^ through the secretion of glia stimulating factors, or by extracellular (or CSF) Tau.

Importantly, depleting T cells in TE4 mice resulted in lower microglial MHC class II and CD11c expression, reduced p-Tau immunoreactivity and brain atrophy, and improved short-term memory. Blocking immunoregulation of T cells (PDCD1-PDL1 signaling) in TE4 mice through repeated anti-PD-1 antibody administration increased the percentage of immune suppressive regulatory T cells, but did not affect the percentage of effector T cells. However, at the age of 9.5 months, the anti-PD-1 treatment achieved a reduction in p-Tau and brain atrophy. These data present compelling evidence that targeting T cells and modulating immune regulation could be a potential therapeutic approach in Tau-mediated neurodegeneration.

In summary, Chen et al. provided important evidence that both innate and adaptive immune systems are involved in mediating neurodegeneration in AD and tauopathies as a response to pathological Tau alterations in the brain. Their findings highlight the crosstalk between microglia and T cells in the development and the progression of Tau pathology and the associated neurotoxicity, suggesting immune modulation as a new line of treatment strategy in these diseases. A deeper understanding of how the immune modules communicate with one another, how Tau, but not amyloid-β, instructs disease-associated glia and T cell actions, and whether other glial cell types, i.e., astrocytes and oligodendrocytes, are involved will be of paramount importance to develop such therapeutic approaches.
